# Approach and Management of Hypertension After Kidney Transplantation

**DOI:** 10.3389/fmed.2020.00229

**Published:** 2020-06-16

**Authors:** Ekamol Tantisattamo, Miklos Z. Molnar, Bing T. Ho, Uttam G. Reddy, Donald C. Dafoe, Hirohito Ichii, Antoney J. Ferrey, Ramy M. Hanna, Kamyar Kalantar-Zadeh, Alpesh Amin

**Affiliations:** ^1^Division of Nephrology, Hypertension and Kidney Transplantation, Department of Medicine, Harold Simmons Center for Kidney Disease Research and Epidemiology, University of California Irvine School of Medicine, Orange, CA, United States; ^2^Nephrology Section, Department of Medicine, Tibor Rubin Veterans Affairs Medical Center, VA Long Beach Healthcare System, Long Beach, CA, United States; ^3^Section of Nephrology, Department of Internal Medicine, Multi-Organ Transplant Center, William Beaumont Hospital, Oakland University William Beaumont School of Medicine, Royal Oak, MI, United States; ^4^Division of Nephrology, University of Tennessee Health Science Center, Memphis, TN, United States; ^5^Methodist University Hospital Transplant Institute, Memphis, TN, United States; ^6^Division of Transplant Surgery, Department of Surgery, University of Tennessee Health Science Center, Memphis, TN, United States; ^7^Division of Nephrology and Hypertension, Department of Medicine, Comprehensive Transplant Center, Northwestern University Feinberg School of Medicine, Chicago, IL, United States; ^8^Division of Transplantation, Department of Surgery, University of California Irvine School of Medicine, Orange, CA, United States; ^9^Department of Medicine, University of California Irvine School of Medicine, Orange, CA, United States

**Keywords:** antihypertensive medications, bilateral native nephrectomy, blood pressure targets, cardiovascular diseases, kidney transplantation, post-kidney transplant hypertension, native renal sympathetic denervation, 24-h blood pressure monitoring

## Abstract

Hypertension is one of the most common cardiovascular co-morbidities after successful kidney transplantation. It commonly occurs in patients with other metabolic diseases, such as diabetes mellitus, hyperlipidemia, and obesity. The pathogenesis of post-transplant hypertension is complex and is a result of the interplay between immunological and non-immunological factors. Post-transplant hypertension can be divided into immediate, early, and late post-transplant periods. This classification can help clinicians determine the etiology and provide the appropriate management for these complex patients. Volume overload from intravenous fluid administration is common during the immediate post-transplant period and commonly contributes to hypertension seen early after transplantation. Immunosuppressive medications and donor kidneys are associated with post-transplant hypertension occurring at any time point after transplantation. Transplant renal artery stenosis (TRAS) and obstructive sleep apnea (OSA) are recognized but common and treatable causes of resistant hypertension post-transplantation. During late post-transplant period, chronic renal allograft dysfunction becomes an additional cause of hypertension. As these patients develop more substantial chronic kidney disease affecting their allografts, fibroblast growth factor 23 (FGF23) increases and is associated with increased cardiovascular and all-cause mortality in kidney transplant recipients. The exact relationship between increased FGF23 and post-transplant hypertension remains poorly understood. Blood pressure (BP) targets and management involve both non-pharmacologic and pharmacologic treatment and should be individualized. Until strong evidence in the kidney transplant population exists, a BP of <130/80 mmHg is a reasonable target. Similar to complete renal denervation in non-transplant patients, bilateral native nephrectomy is another treatment option for resistant post-transplant hypertension. Native renal denervation offers promising outcomes for controlling resistant hypertension with no significant procedure-related complications. This review addresses the epidemiology, pathogenesis, and specific etiologies of post-transplant hypertension including TRAS, calcineurin inhibitor effects, OSA, and failed native kidney. The cardiovascular and survival outcomes related to post-transplant hypertension and the utility of 24-h blood pressure monitoring will be briefly discussed. Antihypertensive medications and their mechanism of actions relevant to kidney transplantation will be highlighted. A summary of guidelines from different professional societies for BP targets and antihypertensive medications as well as non-pharmacological interventions, including bilateral native nephrectomy and native renal denervation, will be reviewed.

## Introduction

Barring contraindications, kidney transplantation is the treatment of choice for advanced chronic kidney disease (CKD) and end-stage renal disease (ESRD) (1). Survival benefit and quality of life are significantly improved after a successful kidney transplantation with renal allograft function. Since the introduction of calcineurin inhibitors (CNI) in the 1980's, short-term renal allograft survival has greatly improved, but there has been no significant effect on long-term renal allograft survival (2, 3).

Several immunological and non-immunological causes contribute to long-term renal and patient survival outcomes. Similar to non-transplant patients, cardiovascular diseases (CVD) remain the leading cause of morbidity and mortality in kidney transplant recipients (4). Hypertension (HTN) is a usual finding in this population and one of the most common risk factors for CVD (5).

This article will review the pathogenesis of post-transplant HTN, including transplant renal artery stenosis (TRAS) and differing management options based on the etiology of hypertension in different clinical transplant recipient scenarios. Determining when non-pharmacological interventions, including transplant renal artery angioplasty and/or stenting, bilateral native nephrectomy, and native renal denervation (RDN), are appropriate will also be discussed.

## Epidemiology of Post-Transplant Hypertension

Depending on the definition and methods of blood pressure (BP) measurement utilized, the prevalence of post-transplant HTN has been widely reported, and it has generally increased over time. This greater incidence of post-transplant hypertension maybe related to the introduction of cyclosporine (CsA) (6–8). A study in Spain looked at patients transplanted in three different years (1990 vs. 1994 vs. 1998) and noted a progressive increase in the incidence of post-transplant HTN with subsequent years for all those three periods. The number of antihypertensive medications required in more recent transplants also increased compared to patients who were transplanted earlier (9). Overall prevalence of post-transplant HTN has ranged from 24 to 90% (5, 8–19).

## Definition of Post-Transplant Hypertension

Post-kidney transplant HTN can be defined as a persistently elevated BP or normotension with use of antihypertensive medications after successful kidney transplantation. However, the main question that remains is what is a normal BP level? Different studies have defined post-transplant HTN with different cutoff levels for systolic and diastolic blood pressure (SBP and DBP) and different requirements for the use of antihypertensive medications. Table 1 summarizes the details of these studies (8, 9, 16–18).

**Table 1:** Summarized definitions of post-transplant hypertension from studies specifically examining the prevalence of post-transplant hypertension.

**References**	**Incidence or prevalence**	**Definition**	**Study design**	***n***	**Mean time since transplantation (range)**
Budde et al. (16)	Incidence 77.3% [81.6% persistent HTN (HTN both pre- and post-transplantation) and 18.4% post-transplant HTN (normotension during pre-transplantation but HTN post-transplantation)]	>150/90 or using antihypertensive medications except the single use of diuretics	A single-center cross-sectional study of patients with stable graft function (>3 months) Mean of ≥5 consecutive BP records Sphygmomanometer in the sitting position	409 patients (64.5% had pre-KTx HTN and 35.5% had pre-KTx normotension) Mean age 47 ± 1 (19–68) years	45 ± 2 months (3–204)
Malek-Hosseini et al. (17)	Incidence 60% [68% persistent HTN (HTN both pre- and post-transplantation) and 32% post-transplant HTN (normotension during pre-transplantation but HTN post-transplantation)]	145/95 or required antihypertensive medication	A single-center study	84 patients (67.9% had pre-KTx HTN and 32.1% had pre-KTx normotension) Mean age at transplantation was 33.5 ± 11.3 years (range 11–58)	34 ± 22.6 months (3–93)
Zeier et al. (8)	Prevalence 90%	>140/90 mmHg or antihypertensive treatment	150 kidney transplants recipients in outpatient clinic with a median follow-up of 3.8 years		
Kasiske et al. (18)	Incidence 50–80%	≥140/90 mmHg	Clinical Practice Guidelines by searches conducted using Medline and pertinent bibliographies and an electronic database used to collate references, but no systematic data extraction or synthesis Experts' opinions		
Campistol et al. (9)	≥80% 3 years post-KTx 85% 5 years post-KTx	SBP ≥140 and/or DSP ≥90 and/or treated with antihypertensive medications	Data from the Spanish Chronic Allograft Nephropathy Study	3,365 adult kidney transplant recipients	

*DBP, diastolic blood pressure; HTN, hypertension; KTx, kidney transplantation; SBP, systolic blood pressure*.

**Figure 1:**
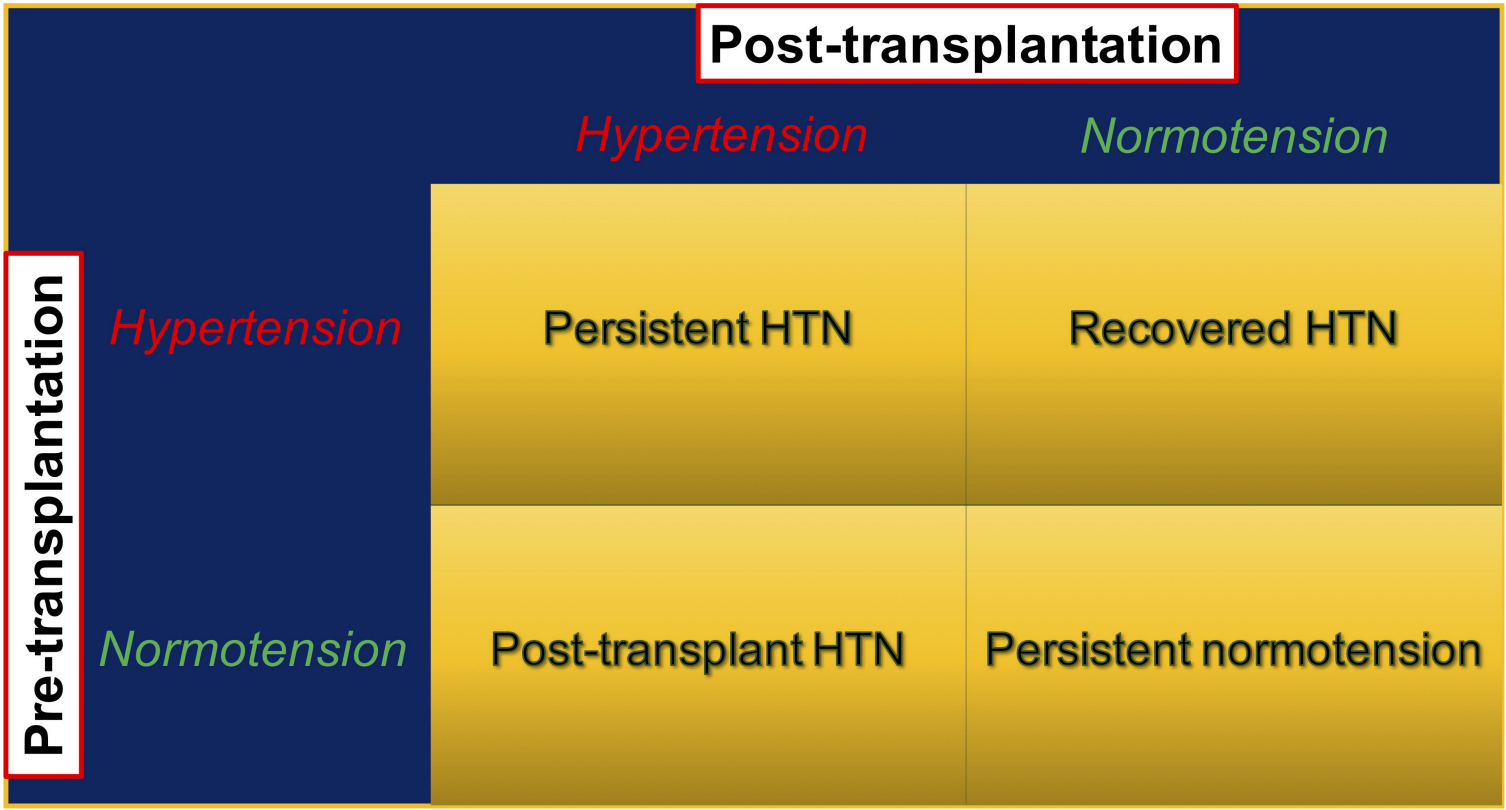
Post-kidney transplant hypertension stratified by presence and absence of pre-transplant hypertension. HTN, hypertension; KTx, kidney transplant.

In addition to the defining normal BP levels, the presence or absence of HTN during the pre-kidney transplant period may further categorize kidney transplant recipients into four groups: persistent HTN, recovered HTN, persistent normotension, and post-transplant HTN. Persistent HTN occurs in patients with HTN both in the pre- and post-transplant periods, whereas patients with recovered HTN have HTN only during the pre- but not the post-transplant period. Persistent normotensive patients have no history of HTN preceding transplant and remain normotensive post-transplant. Post-transplant HTN requires developing *de novo* HTN after kidney transplant (Figure 1). Malek-Hosseini et al. (17) reported the incidences of persistent HTN, recovered HTN, persistent normotension, and post-transplant HTN as 40, 28, 13, and 19%, respectively. In this review, post-kidney transplant HTN refers to persistent and post-transplant (*de novo*) HTN unless otherwise specified.

Isolated forms of HTN both systolic and diastolic still occur after kidney transplantation. The European Society of Hypertension and the European Society of Cardiology guidelines defined isolated systolic HTN in the general population as SBP ≥140 and DBP <90 mmHg (20). This is the most common phenotype of HTN in elderly patients (21). Linear increase in systolic and diastolic BP occurs with age until the fifth or the sixth decades of life when SBP continues increasing, but DBP tends to decrease (22). Pathogenesis of isolated systolic HTN involves in both intrinsic alterations resulting from normal aging process accompanied by development of modifiable risk factors leading to increased arterial stiffness (23).

Alternatively, diastolic HTN is defined as DBP of ≥90 mmHg, with a SBP <140 mmHg, and is more common in younger, sedentary individuals with a higher body mass index (BMI) (24).

A recent large randomized controlled clinical trial of blood pressure management in non-diabetic patients (SPRINT) demonstrated cardiovascular (CV) benefits of tighter BP control (25) leading to new BP guidelines and re-defined HTN for the general population as systolic blood pressure (SBP) >130 or DBP >80 mmHg (26, 27). Although there has been a change in the definition of HTN in the non-transplant population, the definition of HTN in kidney transplant recipients remains controversial, and hard outcomes related to BP levels are still limited. A recent 2017 American College of Cardiology/American Heart Association (ACC/AHA) guidelines recommend a target BP of <130/80 mmHg (26, 27). Until there is stronger evidence of an association between BP level and outcomes in kidney transplant recipients, a BP ≥130/80 mmHg may be a reasonable definition for HTN in this population.

## Pathogenesis of Post-Transplant Hypertension

**Figure 2:**
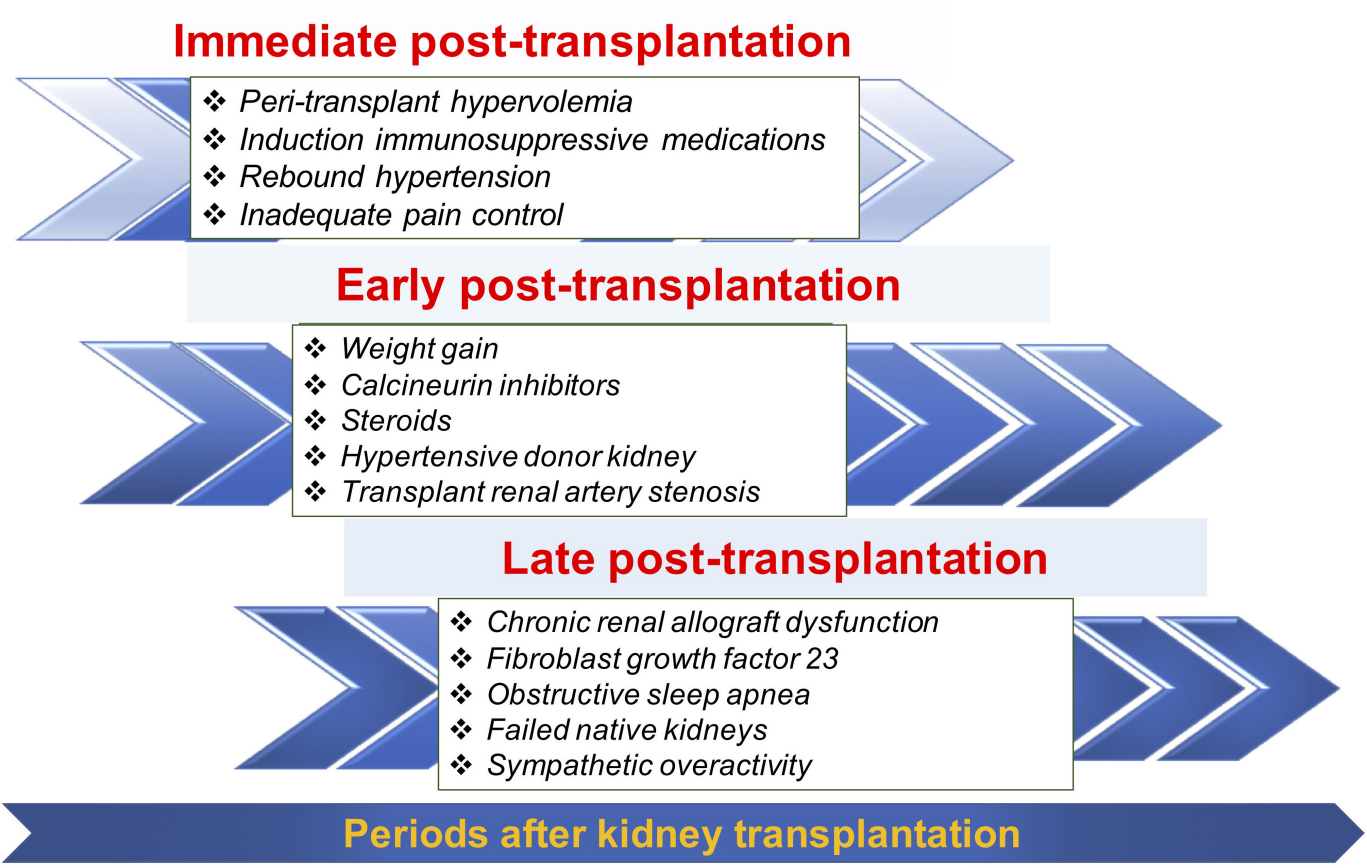
Selected common factors contributing to post-transplant hypertension during three different periods.

The change in prevalence of post-transplant HTN across different post-transplant periods may reflect the differences in pathogenesis of post-transplant HTN over time (Figure 2). Identifying when post-transplant HTN first occurred can narrow the differential diagnosis for the etiology of post-transplant HTN and lead to tailored therapy.

### Immediate Post-transplant Period

During this time, post-transplant HTN is generally a result of external factors like transplant surgery, IV fluids, and high doses of steroids.

#### Peri-transplant Hypervolemia

IV fluid given during surgery and in the immediate post-operative period can lead to hypervolemia, particularly in patients with delayed graft function (DGF). A single-center cross-sectional study showed that the prevalence of hypervolemia measured by multifrequency bioimpedance analysis for extracellular fluid in stable kidney transplant recipients was 30%, and up to 5% had severe hypervolemia. This study showed that hypervolemia was significantly associated with elevated systolic, diastolic, and mean arterial pressures (28). Although this study enrolled 123 kidney transplant recipients with a median duration of enrollment at 5 years post-transplant, hypervolemia most commonly occurred during the immediate post-transplant period, and weight gain above the pre-transplant estimated dry weight was associated with HTN.

Several indicators can be used to estimate volume status. These include conventional measurements such as BP, heart rate, urine output, central venous pressure, and pulmonary artery pressure as well as non-conventional measurements such as intraoperative transesophageal echocardiography and non-invasive dynamic cardiac output technology, e.g., pulse contour analysis, pulse wave transit time, thoracic electrical bioimpedance/bioreactance, and carbon dioxide rebreathing technologies (29, 30). Although many of the non-conventional measurements are useful for volume assessment, they are not readily available during peri-transplant period and conventional measurements remain the standard in clinical practice.

#### High-Dose Steroids

High-dose steroids are commonly used immunosuppressive medications during the peri-transplant period. The incidence of steroid-induced HTN during the immediate post-transplant period is unknown. The mechanism of steroid-induced HTN is unclear, but it may result from alterations in intrinsic pressor response leading to arterial vascular resistance (31). Since a steroid dose of more than 20 mg of prednisone per day is the threshold for having HTN (32), high-dose IV steroids can contribute to HTN during immediate post-transplant period.

#### Rebound Hypertension

ESRD patients commonly have uncontrolled HTN and require several medications to control their BP. During the immediate post-transplant period, it is common practice to hold some if not all of the pre-transplant BP medications in an effort to avoid early hypotension. However, this abrupt discontinuation of antihypertensive therapy can lead to rebound hypertension, an elevated BP above pretreatment levels, as a result of sympathetic overactivity. Beta-adrenergic agonists, clonidine (both oral and transdermal forms) (33), and beta-blockers are commonly associated with this phenomenon, particularly when abruptly stopped. Geyskes et al. demonstrated that almost all patients who took clonidine 900 mcg per day for more than 1 month developed hypertension after the medication was discontinued. Sympathetic overactivity without renin angiotensin system mediation plays a major role in the mechanism of rebound HTN from clonidine (34).

Similar to clonidine, rebound HTN from beta-blockers causes elevated BP and heart rate. It also leads to cardiac events including angina, myocardial infarction, or sudden cardiac death in patients with underlying coronary artery disease (CAD) (35–39). Increased sympathetic activity associated with upregulated adrenergic receptors in individuals taking beta-blockers is thought to be the mechanism of beta-blockers rebound HTN (36, 38, 40). Given the cardiac risk with peri-operative beta-blocker withdrawal (41, 42), the 2014 ACC/AHA guidelines give a class I recommendation with B level evidence for the continuation of beta-blockers during the peri-operative period in patients who chronically use them (43).

#### Inappropriate Pain Management

Acute pain is associated with elevated BP via sympathetic nervous system activation, leading to increases in peripheral vascular resistance, heart rate, and stroke volume. In addition stimulation of the neuroendocrine system via hypothalamic-pituitary-adrenal axis leads to pain-induced HTN (44). Inadequate peri-operative pain control is one of the most common causes of HTN immediately post-transplant. Opioid analgesic is commonly used for pain management during immediate post-transplant period. Non-steroidal anti-inflammatory drugs (NSAID) can provide effective pain relief, but should be avoided in the post-transplant period because of their negative renal effects, including reduced renal plasma flow particularly in patient without establishing renal allograft function or those with DGF.

### Early Post-transplant Period

There has not been a period after kidney transplantation that has been classically defined as the early post-transplant period. However, we found that systolic HTN (≥140 mmHg) occurred at the mean duration of 26–50 weeks after kidney transplantation (45), while the baseline renal allograft function and stable dose of maintenance immunosuppressive medications are generally reached around 3–6 months post-transplant. Therefore, we define the early post-transplant period for this review as the time between 24 and 52 weeks post-transplantation. During which time several factors contribute to HTN.

#### Weight Gain

Between 6- and 12-months post-transplantation, weight gain commonly occurs (46, 47). The mean weight gain and increase in BMI at 1 year post-transplant is 6.2 ± 10.7 kg and 2.1 ± 3.8 kg/m^2^, respectively (47). Obesity (BMI ≥ 30 kg/m^2^) after kidney transplantation is significantly associated with post-transplant HTN (48). Positive fluid intake from intra-and post-operative IV fluid is a common cause of weight gain during the early post-transplant period (49). The amount of sodium in the IV fluid can contribute to HTN. However, after regaining renal allograft function, urinary excretion of the fluid gain can mobilize sodium and water. This can partly help to control BP.

#### Calcineurin Inhibitors

The prevalence of HTN in kidney transplant recipients is between 70 and 90% (50, 51), which is greater than the prevalence during pre-CNI era of 40–50% (52, 53). There are two main mechanism of CNI-induced post-transplant HTN resulting from interfering vascular tone and renal sodium transport handling.

##### Altered vascular tone

Both increased vasoconstriction and impaired vasodilation contribute to CNI-induced post-transplant HTN; although, the latter is thought to be the main mechanism (54).

Renal vasoconstriction. Renal vasoconstriction is mediated by endothelin, a vasoconstrictor, rather than angiotensin II, as captopril does not prevent CsA-induced renal vasoconstriction (55). However, the vasoconstrictive effect on renal or systemic vasculature remains unclear (56). There are inconsistent reports on the effect of angiotensin II on blood vessels. CsA causes local effect on smooth muscle cells by increasing the number of angiotensin II type 1 receptors resulting in vasoconstriction (57).

Renal vasodilation. Impaired vasodilation is a result of CNI-induced reduction of nitric oxide, a vasodilator. CNIs inhibit inducible nitric oxide synthase in vascular smooth muscle cells (58).

##### Increased renal sodium transport handling

Sympathetic nervous system activation. CsA causes sympathetic excitation and subsequent sodium retention (59). There is a link between CNI-induced HTN and phospho-protein synapsin found on microvesicles in renal sensory nerve endings (60).

With-No-K(Lys)—STE20/SPS1-related proline/alanine-rich kinase—Sodium Chloride Cotransporter (WNK-SPAK-NCC) pathway. CNI induces salt-sensitive HTN via activation of the WNK- SPAK-NCC pathway similarly to a rare genetic form of HTN, called familial hyperkalemic hypertension (FHHt, also called Gordon syndrome or pseudohypoaldosteronism type 2) (61). FHHt results from a loss-of-function mutation of WNK kinases that activate NCC (62) and manifests as hyperkalemic hypertension with a non-anion gap metabolic acidosis and hypercalciuria (61). In normal circumstances, calcineurin, a phosphatase, inhibits some kinases, including the kinases WNK3, WNK4, and SPAK in the distal convoluted tubule (DCT), which interact to phosphorylate and activate NCC (54, 63). CNI inhibits calcineurin and leads to phosphorylation and activation of WNK and SPAK kinases and NCC. Therefore, sodium and chloride reabsorption in the DCT is increased and salt-sensitive HTN occurs. Low fractional excretion of chloride supports increased NCC activity (64), and a decreased plasma aldosterone level is consistent with volume expansion (54). From a mechanistic standpoint, thiazide diuretics should be effective for CNI-induced HTN (54, 65).

#### Steroids

Since steroids can cause HTN, steroid avoidance or withdrawal (SAW) maintenance immunosuppressive medication regimens can be considered. However, the effect of SAW on post-transplant HTN has yielded conflicting data. Curtis et al. (10) showed that the prevalence of HTN decreased in patients taking alternate-day steroid therapy. A systematic review and meta-analysis revealed that steroid avoidance or withdrawal significantly decreases CV outcomes including HTN but increases risk of acute rejection (66). It is common for steroids to be reintroduced after a diagnosis of acute rejection in recipients who were initially managed with a SAW regimen. A randomized control trial, however, demonstrated no difference in blood pressure change between alternative day and daily prednisone (67). SAW protocols should be considered in select patients, specifically those who would be at greater risk for CV outcomes but be immunologically lower risk of rejection.

#### Hypertensive Donor Kidney

Salt intake can lead to water retention and HTN, especially in salt-sensitive individuals (68). In addition, several animal studies have demonstrated a renal role in the development of HTN (69–73). Kidney transplantation from idiopathic hypertensive rat donors to genetically normotensive recipients led to post-transplant HTN resulting from decreased renal salt excretion. On the other hand, transplantation from genetically normotensive rat donors to hypertensive rats with pre-transplant bilateral native nephrectomy led to normotension after transplantation (74).

A study in kidney transplant recipients who had undergone native nephrectomy before kidney transplantation from normotensive donors found that all recipients were normotensive post-transplant without need for antihypertensive therapy (75). Another study in kidney transplant recipients from normotensive families found a net increase in antihypertensive requirement antihypertensive requirement after kidney transplantation when kidneys came from donors with hypertensive families compared to those receiving kidneys from donors with normotensive families. However, in recipients with familial HTN, kidney transplantation from any type of kidney did not influence the prevalence of post-transplant HTN (76). In addition, among kidney transplant recipients with no family history of HTN, patients receiving kidneys from donors with family history of HTN required 10 times greater antihypertensive medication requirement as compared to those who receive kidneys from donors without a family history of HTN (77). This all suggests a role for kidney and genetic kidney disease in developing HTN.

#### Transplant Renal Artery Stenosis (TRAS)

Vascular complications are one of the major causes of poorer transplant outcomes. TRAS is a well-recognized and common vascular complication, which leads to worse renal allograft function and CV complications including post-transplant HTN. It is crucial to recognize this early since treatment cause reverse those negative outcomes. Approximately 1–5% of post-transplant HTN is secondary to TRAS (78, 79). However, because of differing definitions applied in studies, the incidence of TRAS was reported to increase from 1 to 23% (80). Wong et al. (81) reported that the prevalence of TRAS increased from 2.4 to 12.4% after the introduction of color Doppler ultrasonography (CDU) in 1985, likely related to improved detection with non-invasive testing. TRAS may occur at any time after kidney transplantation but is generally diagnosed between 3 and 24 months post transplantation (82–84). Unlike renal artery stenosis (RAS) in non-transplant patients, pathogenesis of TRAS is complex and involves non-immunological and immunological factors (81, 85). The non-immunological factors include vascular damage at the time of surgical anastomosis between the donor renal artery and recipient artery as well as the presence of native vascular diseases in both donor and recipient arteries (86). Because the recipient iliac artery, not the abdominal aorta, is the most common vascular target for donor renal artery anastomosis, the connection between these smaller arteries may be predisposed to narrowing and subsequent development of TRAS physiology (87). Fibromuscular dysplasia is not a common cause of RAS in transplant patients. Immunological factors leading to vascular endothelial dysfunction can cause TRAS. Other transplant-related risk factors have been reported including cytomegalovirus (CMV) infection (88). Similar to non-transplant patients, atherosclerotic disease can cause TRAS, but pathogenesis of atherosclerotic TRAS may be different. Atherosclerotic TRAS is unlikely to occur in the early post-transplant period unless there is pre-existing donor and/or recipient atherosclerotic diseases (89). In addition to traditional risk factors for atherosclerotic disease, some immunologic factors may be at play in atherosclerotic TRAS. For example, diffuse stenosis may suggest immune-mediated vascular endothelial injury (90). Moreover, similar histological findings between vascular rejection and stenotic transplant renal arteries (82, 91) as well as an association between post anastomotic TRAS and *de novo* class II donor-specific antibodies (92) raise the possibility of immunologic contribution to atherosclerotic TRAS.

Symptoms and signs of TRAS are non-specific; however, common clinical clues that should lead to a work-up for TRAS are unexplained worsening renal allograft function or uncontrolled HTN (79). Since renal hypoperfusion causes increased renin, angiotensin, and aldosterone, salt retention can lead to peripheral edema, congestive heart failure, and flash pulmonary edema. Notably, paradoxical normotension or hypotension can be seen with use of high-dose diuretics and/or angiotensin-converting enzyme inhibitors (ACEI) or angiotensin II receptor blockers (ARB) (93). Bruits over transplant renal allografts site are common but non-specific. Bruits may be related to other causes like arteriovenous fistula (AVF) in a kidney after biopsy (94).

Several imaging studies can be used to diagnose TRAS. CDU is often the initial imaging study used because it is non-invasive, widely available, and relatively inexpensive. However, the image quality and interpretation are dependent on ultrasonographer technique and experience. Peak systolic velocity (PSV) of the main renal artery and poststenotic intrarenal arterial resistive index (RI) are used to determine and grade the severity of TRAS (79). However, since the diagnostic value of CDU is operator dependent, other imaging modalities may be utilized to verify the diagnosis. Renal artery computed tomography (CT) or magnetic resonance angiography (MRA) should be utilized to further delineate or confirm the diagnosis. However, the risk of contrast-induced nephropathy (CIN) and nephrogenic systemic fibrosis would need to be considered and may limit the use of these imaging studies. Renal artery angiography remains the gold standard diagnostic test for TRAS, but it is invasive and can lead to CIN. Carbon dioxide (CO_2_) angiography can mitigate some of the risk of CIN, but, in most cases, small amounts of IV contrast are still required to attain sufficiently detailed images.

Three therapeutic options for TRAS are pharmacological therapy alone or pharmacologic therapy in addition to renal artery angioplasty with stenting or surgical revascularization (90).

For pharmacological therapy, the pathophysiology of TRAS is similar to that of bilateral RAS in the non-transplant population. A decrease in renal blood flow to a transplant renal allograft causes increased RAAS activation resulting in salt and water retention and subsequent HTN. ACEI or ARB plus diuretics are a likely effective regimen for BP control. However, this pharmacologic therapy is limited by decreasing renal function resulting from decreases in systemic BP leading to reduced renal perfusion and intraglomerular pressure below the limitation of autoregulation. This increases efferent arteriolar resistance mediated by angiotensin II. By blocking the action of angiotensin II, autoregulation is blunted, and GFR is reduced (95). Volume depletion from diuretics may also contribute to rising serum creatinine in these instances. For this reason, it is not common to utilize RAAS and/or diuretics during early post-transplant period. Once baseline renal allograft function is established, however, and there are no contraindications for RAAS and/or diuretics, such as rising serum creatinine, hyperkalemia, or volume depletion, medical therapy may be utilized to control blood pressure (79). Statins and acetylsalicylic acid may also be part of pharmacological therapy although there is no clear evidence for these use specifically in TRAS (79).

Patients with worsening serum creatinine and/or uncontrolled HTN attributable to TRAS should undergo renal artery angioplasty with stenting. There is no randomized controlled clinical trial (RCT) comparing the efficacy of angioplasty ± stenting vs. surgical revascularization vs. pharmacological therapy alone in the kidney transplant population. Data from non-transplant patients from 4 RCT (96–99) did not show the benefit of angioplasty on BP control, and 4 RCT (97, 99–101) did not demonstrate better renal outcomes. However, several observational studies demonstrate highly successful both technical and clinical outcomes (88–100 and 65–94%) with varied procedure-related complications (0–25.5%) in kidney transplant recipients (102–105). Long-term renal allograft and patient survival with up to 21 years of follow-up is not different between patient with TRAS undergoing percutaneous angioplasty or stent placement and patients that without TRAS (105). A single study showed that both immediate and long-term success rate were lower in angioplasty compared to surgical revascularization; however, the former was still a preferred procedure when TRAS is recent, linear, and distal. Whereas, surgical revascularization is performed primarily in individuals with kinking and proximal TRAS (106). There are several surgical techniques, including resection and revision of the anastomosis, saphenous vein bypass graft of the stenotic segment, localized endarterectomy, and excision/reimplantation of the renal artery (90, 107). Generally, surgical revascularization is reserved for cases of unsuccessful angioplasty. Long-term renal allograft function and survival with a mean follow-up of 9.8 ± 2.1 years has demonstrated the efficacy and safety of surgical revascularization (108).

### Late Post-transplant Period

Apart from the above-discussed factors contributing to HTN in the early post-transplant period, some factors may contribute to HTN in the late post-transplant period.

#### Chronic Renal Allograft Dysfunction

A causal relationship between chronic renal allograft dysfunction and HTN has not been proven but follows on from a logical examination of pathophysiology. Renal allograft injury—both acute and chronic—is associated with HTN. Common causes of renal allograft injury include acute allograft rejection—both acute antibody-mediated—and acute cellular rejection. Chronic renal allograft injury results from persistent antibody-mediated rejection, interstitial fibrosis/tubular atrophy, thrombotic microangiopathy, and recurrent glomerular disease in the renal allograft (19). An animal study in rats showed that HTN alone led to chronic allograft nephropathy (CAN). This study utilized rats with deoxycorticosterone acetate and salt-induced hypertension and compared them to normotensive rats. The hypertensive rats had higher proteinuria, smooth muscle cell-growth factors, platelet-derived growth factor (PDGF), tubular cell expression of proliferating cell nuclear antigen, extracellular matrix deposition, and presence of class I and II major histocompatibility complex (MHC). This suggests that HTN and immunologic factors affect the expression of growth factors in renal allografts and may be the cause of chronic renal allograft dysfunction (109).

#### Fibroblast Growth Factor (FGF) 23

After kidney transplantation, FGF23 levels decrease. The rate at which FGF23 levels normalize after transplantation is dependent on shorter dialysis vintage before kidney transplantation and the time to normalization of renal allograft function including mineral metabolism (110, 111). FGF 23 is a known independent risk factor of renal allograft loss, cardiovascular and all-cause mortality in kidney transplant recipients (112, 113). The relationship of FGF 23 levels in the pre- and post-kidney transplant period to BP level or development of HTN after kidney transplantation is unknown. However, given that a higher level of FGF23 is associated with increased SBP and DBP as well as an incident HTN in 1,758 non-hypertensive young adults without CKD or CVD (114), this association may hold true for the transplant population. Despite this, further studies are necessary.

#### Obstructive Sleep Apnea

Prevalence of obstructive sleep apnea (OSA) in kidney transplant recipients varies with the severity of OSA. The prevalence of mild, moderate, and severe OSA in kidney transplant recipients participating in Sleep disorders Evaluation in Patients after kidney Transplantation (SLEPT) Study who had a mean eGFR of 52 ± 19 ml/min/1.73 m^2^ was 18, 11, and 14%, respectively. In this study, kidney transplant patients with OSA required a significantly higher number of antihypertensive medications and tended to have higher SBP as compared to non-OSA patients (115).

Similar to the non-transplant population, risk factors for OSA in kidney transplant recipients are male gender, obesity, use of hypnotic drugs, presence of severe comorbidity (e.g., heart disease, cerebrovascular disease, peripheral vascular disease, diabetes mellitus), and impaired kidney function (116). There is a relationship between HTN, CKD, and OSA, and they have common risk factors and pathophysiology, including increased sympathetic activity, endothelial dysfunction, increased inflammatory markers, hyperaldosteronism, and chronic volume overload. In addition, these three conditions are associated with CV risks, morbidity, and mortality (117). If any of these three diseases is uncontrolled or progress the other two conditions tend to worsen or become more difficult to manage. Therefore, appropriate management of OSA is an essential component of the antihypertensive therapy in kidney transplant recipients who later develop renal allograft dysfunction especially with resistant HTN. Moreover, kidney transplant recipients who have risk factors for OSA should undergo early screening (115, 118) and be appropriately treated.

**Figure 3:**
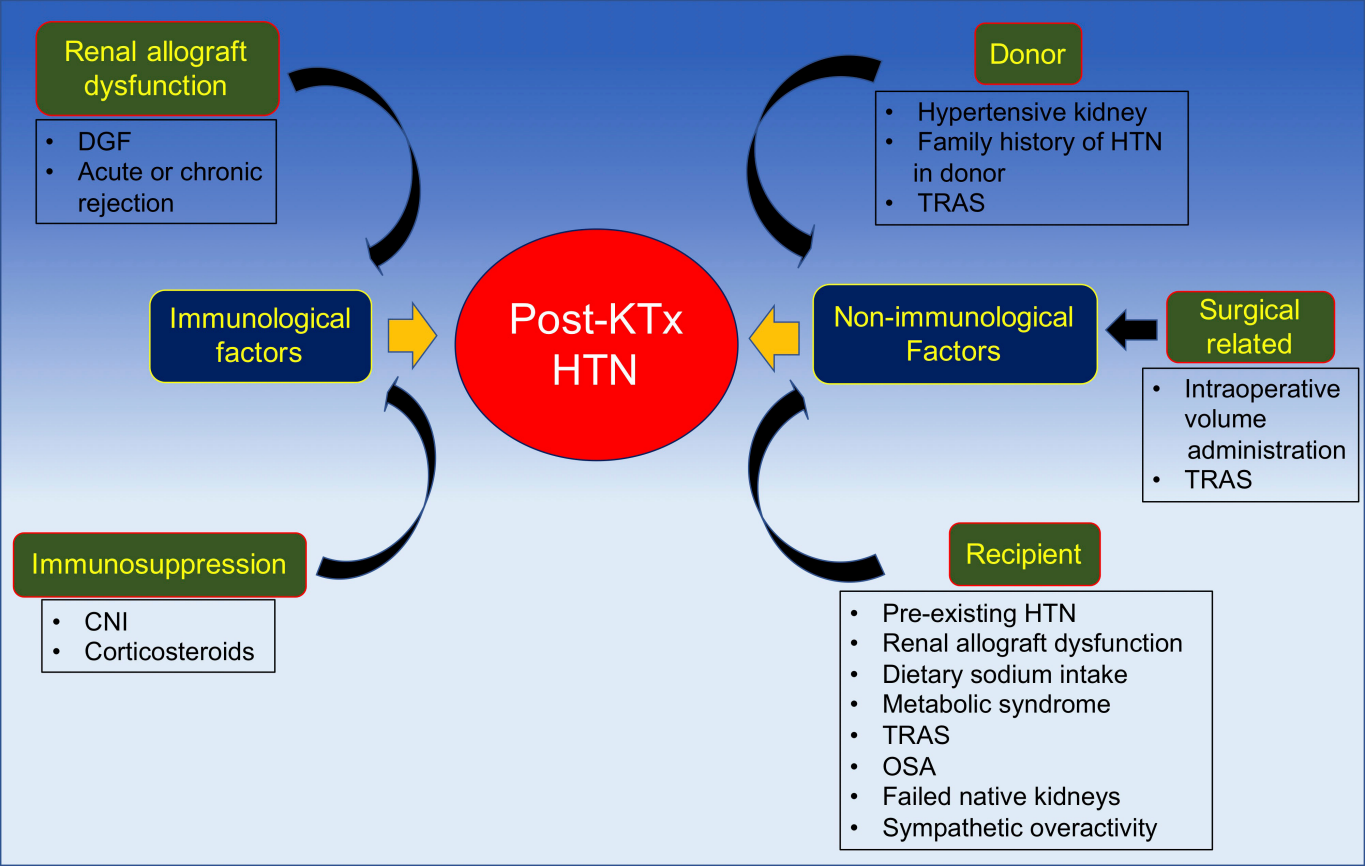
Pathogenesis of post-kidney transplant hypertension divided into immunological and non-immunological factors. CNI, calcineurin inhibitor; DGF, delayed graft function; HTN, hypertension; KTx, kidney transplantation; OSA, obstructive sleep; TRAS, transplant renal artery stenosis.

In summary, the pathogenesis of post-transplant HTN may be categorized by the time period during which hypertension develops after kidney transplantation. Another approach to differentiate the etiologies of post-transplant HTN is by dividing the causes into immunological and non-immunological factors. Immunological factors, as discussed, mainly include renal allograft dysfunction and immunosuppressive medications; while non-immunological factors involve the donor, recipient, and surgical related factors (Figure 3).

## Outcomes of Hypertension After Kidney Transplantation

Pre-transplant BP is associated with renal allograft and patient survival outcomes after kidney transplantation. Very low pre-transplant SBP (<110 mmHg) and DBP (<50 mmHg) are associated with a decrease in renal allograft loss. Specifically during dialysis lower pre- and post-dialysis DBP are associated with better patient survival post-transplantation (119).

During the post-transplant period, elevated BP is associated with poorer renal allograft and patient outcomes. However, as discussed above, various forms of renal allograft injury are also associated with post-transplant HTN. Several studies have demonstrated an association between post-transplant HTN and renal allograft failure (120–122). Opelz et al. (120) conducted a retrospective study of 29,751 kidney transplant recipients followed for over 7 years. Increased post-transplant SBP and DBP were associated with progressively decreased renal allograft function and death-censored chronic graft failure. Another study from the same cohort database examined the association between changes in BP levels at 1- and 3-years post-transplantation and long-term graft outcomes up to 10 years following transplantation. They found that patients with a SBP >140 mmHg at 1 year who were controlled to a SBP ≤ 140 mmHg at 3 years post-transplantation had improved renal allograft outcomes and reduced CV death compared to those with persistent SBP of >140 mmHg both at 1 and 3 years post-transplantation (123).

There is insufficient information about CV and mortality outcomes related to isolated diastolic HTN in kidney transplant recipients, and more studies are required to determine appropriate management targets for isolated diastolic HTN in this population.

### Cardiovascular Outcomes-Related to Post-transplant Hypertension

HTN is one of the most important risk factors for heart failure, particularly heart failure with preserved ejection fraction (HFpEF) (124). Kidney transplant recipients with left ventricular hypertrophy (LVH) or HFpEF have increased morbidity and mortality and are at high risk for cardiac-related events. Both pressure and volume overload contributed to LVH, which is further exacerbated by age, genetic factors, vascular hemodialysis access, dialysis vintage, diabetes, and blood pressure (125).

Renal insufficiency is involved in the pathogenesis of HFpEF (126) and causes salt-sensitive HTN (126, 127). Importantly, a vicious cycle of cardio-renal dysfunction can result from salt and volume overload (126). Therefore, BP control, renal allograft function, and heart function are closely related. Uncontrolled HTN after kidney transplantation leads to structural damage to both the renal allograft and heart eventually resulting in decreased renal and cardiac functions.

## Blood Pressure Measurement

BP is one of the most common vital signs obtained in all clinical settings; however, it may frequently be unreliable due to variation of physiologic response to internal and external stimuli as well as inappropriate BP measurement techniques. Reliable BP measurement should be mandatory in clinical practice and can be standardized with the following definitions: the mean of three non-invasive BP measurements is referred to as office blood pressure (OBP); recording at least twice the daily average of two home blood pressure readings over a minimum of 4 days is referred to as home blood pressure monitoring (HBPM), and 24-h ambulatory blood pressure monitoring (24-h ABPM), which requires wearing an electronic blood pressure measurement device to record and averages multiple readings over a 24 h period (128). Purpose, clinical context, and practicality should be taken into consideration when selecting the appropriate BP measurement methods. The different BP measurement methods provide different information, which can be useful for sorting out the pathophysiology of post-transplant HTN. It is important to be knowledgeable about the different definitions for HTN with each method in order to appropriately manage BP after kidney transplantation.

A 24-h ABPM provides the average of both day and night BP readings. Physiological decreases in nocturnal BP further classifies patients into dippers, non-dippers, and reverse dippers. Lee et al. (129) demonstrated a significant decrease in the nocturnal reduction in SBP (ΔSBP) after kidney transplantation. In addition, decrease in ΔSBP was associated with a lower renal allograft function. In this study, the mean OBP and 24-h ABPM did not change at 1-year post-transplantation when compared to the BP measurements before kidney transplantation. However, they found that the proportion of patients who took antihypertensive medications and the numbers of antihypertensive medications required were significantly decreased after kidney transplantation.

A 24-h ABPM can address and assist with the common misclassification of HTN diagnosed traditionally by OBP or HBPM. Compared with OBP, a 24-h ABPM led to 61% disagreement in diagnosis (58% and 3% due to masked and white-coat HTN, respectively) (130).

Although a 24-h ABPM can provide useful information to diagnose patterns of HTN like white coat and masked HTN, OBP and HBPM are more commonly used in clinical practice. Since transplant renal allografts are very sensitive to BP hemodynamic changes, HBPM appears to be a common utilized method of following BP after kidney transplantation. One study in patients who were 1–10 years after kidney transplantation revealed a higher correlation between a 24-h ABPM and HBPM than 24-h ABPM and OBP (131).

Though elevations in both day and night SBP obtained from 24-h ABPM were associated with risk of declining renal allograft function, and nighttime elevation in SBP exhibited a stronger association (132). An elevated 24-h average SBP was significantly associated with a composite endpoint of graft loss, cardiovascular events and death over a 5-year follow-up period in kidney transplant recipients with diabetes, lower eGFR, proteinuria, young age, and who were female (133). An average (day and night) 24-h DBP between 65 and 80 mmHg was associated with greater long term survival during a 9-year follow-up period after kidney transplantation when compared to those who had average DBP <65 or >80 mmHg (134).

Although HBPM is widely available, better correlated to 24-h ABPM, and superior to OBP when predicting hard outcomes, we used HBPM in conjunction with OBP for our kidney transplant patients to minimize misclassification of HTN diagnosis, monitor possible white coat or masked HTN, and adjust antihypertensive medications.

## Blood Pressure Management

Non-pharmacologic interventions, such as diet, exercise, and stress reduction, should always be part of treatment of HTN. Since the majority of kidney transplant recipients have pre-transplant HTN requiring antihypertensive medications (persistent HTN) and only small number of patients become normotensive without blood pressure medications (recovered HTN), pharmacological intervention remains the cornerstone of BP control in this population. Additionally, other interventions specific to certain etiologies of resistant HTN, such as transplant renal artery angioplasty ± stenting and treatment for OSA, should be implemented. Renal sympathetic denervation of the native kidneys either by bilateral native nephrectomy or catheter ablation is also treatment option for resistant HTN in this population (Table 2). In this review, we focus on antihypertensive medications and briefly review bilateral native nephrectomy and RDN of native kidneys.

**Table 2:** Common interventions for post-kidney transplant hypertension.

**Blood pressure managements**	**Interventions**	**Comments**
Lifestyle modifications	Diet Exercise Stress reduction	Required for all patients
Pharmacological therapy	Antihypertensive medication •Diuretics - Loop - Thiazides •Calcium channel blockers •Beta-blockers •Renin-Angiotensin-Aldosterone System blockade - Angiotensin-converting enzyme inhibitors - ARB, angiotensin II receptor blockers - Mineralocorticoid receptor antagonists •Alpha_1_ antagonists •Alpha_2_ agonists	Choice of medications depending on: •Patients' characteristics, •Tolerability •Medication side effect profiles
Procedural or surgical interventions	Specific treatment modalities •Transplant renal artery angioplasty ± stenting •Continuous positive airway pressure (CPAP) •Bilateral native nephrectomy •Native renal denervation	Etiologies of resistant hypertension •Transplant renal artery stenosis •Obstructive sleep apnea (OSA) •Failed native kidney •Sympathetic overactivity

## Pharmacological Managements

### Use of Antihypertensive Medications

A recent retrospective cross-sectional analysis from a single transplant center in Poland reported the trend of commonly used antihypertensive medications in their kidney transplant recipients over 14 years. They examined antihypertensive therapy data from transplant recipients first outpatient visits from 2001, 2006, 2011, and 2014 (135). Beta-blockers were the most common antihypertensive medication used in this cohort followed by calcium channel blockers. Uses of ACEI, diuretics, and alpha-blockers were about the same. ARB therapy was utilized least. The average number of antihypertensive medications required was reported to be 2.24 ± 1.03–2.55 ± 1.25. This is slightly lower than what has been reported for CKD patients who on average require 3.5 medications (136). The lower number of antihypertensive medications is likely from regaining kidney function after transplantation and improved solute clearance, including salt and volume excretion by renal allograft.

## Blood Pressure Control During Peri-Transplant Period

### Antihypertensive Medications

The majority of advanced CKD and ESRD patients have HTN. Volume overload is the most common cause of uncontrolled BP in ESRD patients (137). In the immediate pre-transplant period, particularly in deceased donor renal transplantation when surgeries are often performed emergently (138), BP can be uncontrolled particularly if the patient was due for dialysis at the time of surgery. Volume-dependent HTN is managed with fluid removal during dialysis. However, it is common practice to remove less fluid from patients who are about to undergo transplant, leaving them with a post-dialysis weight slightly above their established dry weight in an effort to avoid intra- and post-operative hypotension. Additionally, ACEI and ARB are generally held following a transplant. An alpha_2_ agonist like clonidine, however, may need to be continued during peri-transplant period to avoid rebound HTN.

Providers taking care of kidney transplant recipients have different opinions when selecting antihypertensive medications during peri- and post-transplant period. Their decision may mainly be driven by protocols or common practices followed at their kidney transplant centers. Specific information relevant to kidney transplantation should guide the selection of antihypertensive medications, but, as in other scenarios, it is rational to individualize BP management for certain kidney transplant recipients.

### Diuretics

Diuretics are not commonly used as the first line antihypertensive medication in kidney transplant recipients. They may cause volume depletion, electrolyte disturbances, and worsening renal allograft function. However, they are indicated and should not be avoided in certain patients in the peri-transplant period.

### Loop Diuretics

Volume control rather than BP control is the indication for loop diuretics in kidney transplant recipients, especially during immediate and early post-transplant periods. Kidney transplant recipients generally receive peri-transplant IV fluid to keep up with an increased urine output from a new functioning renal allograft. Volume overload presenting with peripheral edema, pulmonary congestion, or HTN may occur when the establishment allograft function lags behind the volume resuscitation provided. Diuretics can be used to control both volume and BP in this common scenario.

There is also evidences that loop diuretics have a vasodilator effect and can decrease edema, congestion, and oxygen requirements. Theoretically they can decrease ischemic renal injury and risk of DGF in kidney transplant recipients. However, despite their common use in clinical practice, there is no consistent data to support using loop diuretic to increase urine output and prevent DGF. In addition, there is no strong evidence showing an association between loop diuretic and improvement in initial or long-term graft function (139). On the other hand, loop diuretic use has been associated with increased risk of UTI during the first 5 years after kidney transplantation (140). This is because there use depletes medullary NaCl gradient (140), which is known to modulate the adaptive and innate immune response (141). Renal medullary myeloid mononuclear cell phagocytes are classified as pro-inflammatory (M1) and reparative/profibrotic (M2) cells. Decreasing M1/M2 ratio from altering renal medullary NaCl gradient with loop diuretics impairs the natural antibacterial host response (142–144).

Furosemide is the most commonly used loop diuretic. It has been used to predicting progression of AKI with the so-called furosemide stress test (FST) in non-transplant patients (145, 146). Loop diuretics can also provide prognostic value in regard to renal allograft function in the immediate post-transplant period. An inadequate response, urine output <350 ml within 4 h, to a single IV furosemide dose of 1.5 mg/kg given 3 h after renal allograft anastomosis predicts increased risk of DGF (146).

Given the wide range of risks and benefits with loop diuretics, clinicians should individualize their use in transplant recipients. Additional RCTs are required to determine the efficacy and safety of loop diuretic in kidney transplant recipients.

### Thiazides

Thiazide diuretics are commonly used antihypertensives in general population. They are however uncommon in the management of kidney transplant recipients because of their metabolic side effects which include hyperglycemia, hyperuricemia, hypercalcemia, and hyponatremia (147, 148).

Thiazides however, may theoretically control CNI-induced HTN. Since CNI-induced HTN is salt sensitive (59, 149) via activation of the WNK-SPAK-NCC pathway (63, 150), thiazides, which inhibit Na-Cl co-transporter, should control CNI-induced HTN. A randomized non-inferiority crossover trial comparing the effects of chlorthalidone and amlodipine in kidney transplant recipients taking tacrolimus showed that chlorthalidone was not inferior to amlodipine in controlling BP (147). Given the vasodilator effect of calcium channel blockers, they are commonly used antihypertensives in kidney transplant recipients intended to counteract the systemic and renal vasoconstrictive effect of CNI via endothelin I (151–153). However, their side effects, including peripheral edema and proteinuria, may make thiazides a suitable alternative for BP control particularly in individuals with peripheral edema (147).

Although loop diuretics are generally favored over thiazide diuretic for volume control, they can lead to additional renal magnesium wasting. Thiazides can be considered in transplant patients who already have other reasons for and often struggle with hypomagnesemia. However, because CNI and loop diuretics act on the common mechanism to lower luminal electro-positivity decreasing paracellular magnesium reabsorption, using a loop diuretic in concert with CNI may not cause further urinary magnesium loss (154). A retrospective study in heart transplant recipients taking CNI showed that the group receiving loop diuretic did not have lower serum magnesium levels or require higher magnesium supplementation compared to the group not receiving loop diuretics (154). Thiazide diuretics, on the other hand, increase serum magnesium when used with CNI. In non-transplant patients without CNI, acute thiazide diuretic use results in increased magnesium reabsorption. However, long-term thiazide diuretic use can result in urinary magnesium wasting when there is concomitant hypokalemia (154). Transplant recipients on CNIs commonly have hyperkalemia, and, therefore, thiazide diuretics may not worsen hypomagnesemia from CNI but instead increase magnesium levels. The previously mentioned study demonstrated that heart transplant recipients receiving thiazide diuretics had higher serum magnesium level and required less magnesium replacement compared to those not on thiazides (154). Therefore, in the setting of volume overload and concomitant hypomagnesemia, thiazide diuretic may be used to mitigate the hypomagnesemic effect of CNI therapy (154).

Thiazides may be considered for kidney transplant recipients with CNI-induced salt-sensitive HTN and hypomagnesemia.

### Mineralocorticoid Receptor Antagonists

Mineralocorticoid receptor antagonists (MCRA) have CV benefits (155, 156) and antiproteinuric effect but are not commonly used antihypertensives in kidney transplant recipients, especially those with impaired renal allograft function. Hyperkalemia is a common side effects of MCRA and it may be worse in kidney transplant recipients who have CNI-induced hyperkalemia.

One pilot study evaluating the antiproteinuric effect of spironolactone 25 mg/day given to 11 kidney transplant recipients whose mean proteinuria was 4.4 ± 1.4 g/day on both ACEI and ARB. Proteinuria was decreased more than 50% with a mean reduction of 85% in nine patients after 6 months on spironolactone. Renal allograft function slightly decreased with GFR, changing from 52 ± 12.7 to 48 ± 14.2 mg/dL, and serum potassium did not significantly increase (4.6 ± 0.4–5 ± 0.62 mEq/L) (157). Another study looked at the safety of eplerenone in kidney transplant recipients with eGFR between 30 and 50 mL/min/ 1.73 m^2^. After 8 weeks of eplerenone 25 mg/d, nine out of 31 patients had serum potassium of >5 mmol/l, and one patient had serum potassium of >5.5 mmol/l. Mean baseline eGFR was 41 (26–59) ml/min/1.73 m^2^ after 8 week of eplerenone therapy and was not significantly different between patients who did and did not develop hyperkalemia (36.0 [95% CI 26.0–53.0] vs. 44.5 [95% CI 26.0–59.0], *p* = 0.17). Having a baseline serum potassium >4.35 mmol/l was associated with increased serum potassium of >5 mmol/l once starting eplerenone (158).

CNIs can cause vascular vasoconstriction via activation of mineralocorticoid receptor in smooth muscle leading to impaired renal allograft function. MCRAs bind mineralocorticoid receptors in principle cells of collecting ducts and are potentially reno-protective by blunting the renal vascular resistance induced by CNI therapy (159).

Spironolactone decreases cardiac left ventricular volume and mass, but studies are required to determine its effect in kidney transplant recipients (160). In addition, there is a lack of evidence in terms of CV and mortality benefits in kidney transplant recipients.

With some evidence for antiproteinuric effect and their established safety in transplant recipients even when in combination with ACEI or ARB, MCRA may be a new option for BP control in individual with CNI-induced HTN and proteinuria.

### Beta-Blockers

The cardioprotective effects and survival benefit of beta-blockers make them a favored medication in the general (161) and ESRD populations (162–165). In kidney transplant recipients, a recent retrospective study from 2001 to 2014 showed that beta-blockers are the most common used antihypertensive medication (135). In addition, Aftab et al. (166) conducted a single center retrospective study in 321 kidney transplant recipients over a 10 ± 4 year follow-up period and found that those who took beta-blockers had a significant survival advantage compared to those who did not. Moreover, the authors showed that beta-blocker has additive effect on ACEI or ARB with greater survival in kidney transplant patients on this combination compared to those who received either medication alone or neither. The possible protective mechanism of beta-blocker is via mitigation of the sympathetic nervous system, which is stimulated in failed native kidneys (167–169). In addition, beta-blockers decrease proinflammatory cytokines, which are known to increase the risk for atherosclerosis (170).

Although beta-blockers provide survival benefit in kidney transplant recipients, they can cause metabolic side effects, including proteinuria, hyperkalemia, and masking of hypoglycemic symptoms. Therefore, caution should be used when using beta blockers in kidney transplant recipients who are at risk to develop these side effects.

### Calcium Channel Blockers

Calcium channel blockers inhibit entry of calcium into vascular smooth muscle cells, resulting in vascular vasodilation (171). Since the vasoconstrictive effect of CNIs leads to post-transplant HTN (172), calcium channel blockers are thought to be an appropriate agent for post-transplant HTN. Theoretically their vasodilatory effect can counteract the vasoconstrictive effect of CNIs and improve BP control (171, 173, 174).

In addition to blood pressure control, calcium channel blockers also prevent post-transplant acute tubular injury (ATI) or DGF. One prospective trial looked at diltiazem use in patients who received deceased donor renal transplantation (DDRT). Participants were randomized to kidneys that had been given diltiazem (Euro-collin's solution (20 mg/l) at time donor nephrectomy; meanwhile, the recipients received a bolus injection of diltiazem (0.28 mg/kg) pre-operatively followed by an infusion 0.0022 mg/min/kg for 2 days before transitioning to oral diltiazem vs. a control group, which did not receive diltiazem but otherwise received the same induction immunosuppression regimen with CsA. The diltiazem group had lower incidence of ATI, higher GFR with primary function, and lower incidences of rejection at 1 month post-transplant. They however had higher CsA level compared to control group. The total CsA required was lower in the diltiazem group, as might be expected, to achieve comparable CsA levels between the two groups. The lower post-transplant ATI in the diltiazem group was postulated to be secondary to lower ischemic damage in the renal allograft and reduced CNI nephrotoxicity (175). A Cochran review of 13 trials with a total of 724 participants also concluded that peri-operative calcium channel blocker significantly decreased incidence of post-transplant ATI and DGF. There was no difference in graft loss, mortality, or requirement for hemodialysis, but there was insufficient evidence to draw conclusions regarding adverse drug reactions (176). There was another randomized placebo control study comparing renal allograft outcomes between patients who received pre-transplant isradipine vs. those who received a placebo. The former group had greater renal allograft function as compared to the latter. However, the rate of DGF and biopsy-proven acute rejection was not different in this study (177).

Although calcium channel blockers provide better renal allograft function, several studies showed no difference in terms of BP control when using verapamil compared to enalapril or doxazosin (178). No difference in GFR, serum creatinine level, protein excretion, or BP when nitrendipine or nifedipine was compared to the placebo (179). Moreover, one retrospective study showed 2.26 time greater risk of ischemic heart disease in kidney transplant recipients who received dihydropyridine calcium channel blockers (180).

The evidence for the benefits of calcium channel blockers appears inconsistent; however, a recent systematic review and meta-analysis of 60 trials including 3,802 patients showed that, compared to placebo or no treatment, calcium channel blockers decreased graft loss [risk ratio (RR) of 0.75, 95% confidence intervals (CI) 0.57–0.99] and increased GFR [mean difference (MD) 4.5 mL/min, 95% CI 2.2–6.7]. Compared to ACEIs, patients treated with calcium channel blockers tend to have a higher GFR; although there is inconclusive data regarding graft loss between these two groups (181). Given the available information, calcium channel blockers remain a preferred antihypertensive agent for kidney transplant recipients barring there are no specific indications for other antihypertensive agents or contraindications to calcium channel blocker therapy.

Adverse drug reactions and drug–drug interaction between calcium channel blockers and other commonly used medications should be taken into consideration. Calcium channel blockers can result in peripheral edema and muscle weakness especially when used in combination with steroids. Gum hyperplasia is also more common when calcium channel blockers are used with CsA (171). Although dihydropyridine calcium channel blockers do not inhibit the cytochrome P450 (CYP) 3A4 isoenzyme, they are metabolized by a CYP3A4 isoenzyme and can compete with CNI. This leads to increase in both calcium channel blocker and CNI exposure levels. Non-dihydropyridine calcium channel blockers inhibit CYP3A4 isoenzyme and significantly increase CNI level.

### Angiotensin-Converting Enzyme Inhibitors (ACEI) and Angiotensin II Receptor Blockers (ARB)

Since proteinuria is a surrogate marker for renal disease, lowering proteinuria is one strategy to slow the progression of CKD. ACEIs and ARBs are antihypertensive agents with known antiproteinuric effect. ACEIs inhibit angiotensin-converting enzyme, which converts renin to angiotensin. ARBs act on angiotensin II receptors and subsequently inhibit RAAS pathway. A systematic review and meta-analysis of 21 clinical trials, including 1,549 patients, revealed no difference in the MAP change between ACEI or ARB group and the control group. Serum potassium was also not different between the two groups. However, the ACEI or ARB group had a decrease in proteinuria, eGFR, and hematocrit when compared to the control group. There was insufficient data regarding the benefits of ACEI or ARB therapy in terms of patient or renal allograft survival (182). In the aforementioned systematic review and meta-analysis, eGFR in the ACEI group was not different from that in the placebo group but was lower than the calcium channel blocker group. A comparison of data regarding graft loss between ACEI and calcium channel blocker therapy was inconclusive (181).

RAAS activation is associated with interstitial fibrosis and tubular atrophy (IF/TA), one of the most common causes of renal allograft loss (183, 184). The ACEI perindopril was shown to prevent cortical interstitial expansion, a marker of fibrosis in CKD patients with biopsy-proven diabetic nephropathy (185). One RCT in kidney transplant recipients who received losartan 100 mg daily within 3 months post-transplant and continued it for 5 years compared to matched placebo controls did not show a benefit of ARB therapy in terms of reduction in a composite interstitial expansion or ESRD from IF/TA (186).

ACEIs and ARBs can lead to regression of LVH in kidney transplant recipients (187, 188), but these same studies did not demonstrate an improvement in all-cause mortality (189). A recent systematic review and meta-analysis demonstrated a survival benefit with ACEI or ARB therapy in kidney transplant recipients but only from pooled cohort studies and not RCTs (190).

Although ACEIs and ARBs provide antiproteinuric effect, they are generally not the drugs of choice during the immediate and early post-transplant periods. This is mainly because they are known to decrease GFR. This side effect is reversible, but it leads to a confusion early on differentiating their role from other causes of renal allograft dysfunction and may lead to unnecessary workups, including invasive investigation, e.g., transplant renal biopsy.

While there is no robust evidence for CV or survival advantages with the use of ACEIs, ARBs, and MCRAs, these antihypertensive medications may be considered. They may be considered in transplant recipients, particularly those with LVH, congestive heart failure, and proteinuria.

### Alpha_1_-Antagonists

Alpha_1_-antagonists are rarely used as the initial or as a single antihypertensive agent in kidney transplant recipients. Although they reduce BP by decreasing peripheral vasoconstriction and theoretically should counteract with vasoconstrictive effect of CNI, there is no evidence that alpha_1_-antagonists provide superior antihypertensive effect or slows progression of renal allograft dysfunction when compared to other antihypertensive medications. A study with long-term follow up in 88 hypertensive kidney transplant recipients who were divided into three groups, verapamil, enalapril, and doxazosin, demonstrated that doxazosin provided the same efficacy in BP control compared with the others while maintaining an excellent safety profile. However, up to 38% of patients in the doxazosin group needed additional antihypertensive medications compared to only 8 and 13% in verapamil and enalapril groups, respectively (178). Alpha_1_-antagonist may have a role as an adjunctive therapy rather than first line antihypertensive agent in transplant recipients.

### Alpha_2_ Agonists

Centrally acting alpha_2_ agonists work on presynaptic alpha_2_ adrenoceptors in the central nervous system and suppress central sympathetic activity (191). Specifically, activation of alpha_2A_ receptors causes a sympatho-inhibitory effect and lowers BP. However, stimulating alpha_2A_ receptors in blood vessels causes a peripheral vasoconstriction (192). On the other hand, alpha_2B_ receptor activation leads to a sympatho-excitatory effect (192).

Two of the oldest alpha_2_ agonists, methyldopa and clonidine, have long been used for BP control (193). Clonidine is currently the most commonly used alpha_2_ agonist. When used as monotherapy, methyldopa is associated with antihypertensive tolerance, edema, and weight gain. Clonidine is also associated with weight gain and progressive resistance with continued use. Chronic use is not associated with sodium and water retention or body fluid volume expansion (193). It instead causes a decrease in exchangeable body sodium and plasma volume, which may be another antihypertensive mechanism of clonidine (194).

Similar to other groups of antihypertensive medications, clonidine has an effect on renal hemodynamics and lowers renal vascular resistance. A study in 13 essential hypertensive patients who received long-term clonidine showed that it lowered plasma renin activity, modulated renal vascular resistance, and subsequently lowered MAP (195). A study of the effect of clonidine on renal hemodynamics was conducted in six kidney transplant recipients who initially received furosemide for 2 weeks before the addition of clonidine titrated to BP control found that GFR and effective renal plasma flow measured by inulin and amino-hippurate sodium clearances was unchanged (196).

Clonidine is an effective antihypertensive medication. Many ESRD patients who are on kidney transplant waiting lists have uncontrolled HTN and are on clonidine in addition to their other antihypertensive agents. However, as previously discussed, rebound HTN is common after discontinuation of clonidine, and patients who are on clonidine prior to kidney transplant tend to have uncontrolled HTN resulting from this rebound phenomenon. In these patients, clonidine is often re-started and be tapered off during early post-transplant period. Therefore, clonidine is rarely used as a single antihypertensive agent following kidney transplantation.

In summary, there is no drug of choice for BP control after kidney transplantation. Several factors are involved in selecting the appropriate antihypertensive medications include immunologic and non-immunologic factors as well as the time after kidney transplantation.

Different from the non-transplant CKD population, beta-blockers and calcium channel blockers are the most frequently used combination in kidney transplant recipients. Beta-blockers provide a cardioprotective effect for kidney transplant patients, who likely have underlying CAD (166). Calcium channel blockers have vasodilatory effect that counteracts the vasoconstrictive effect of CNIs (197). ACEIs and ARBs, on the other hand, are not routinely used antihypertensive medications in kidney transplant recipients. This is especially true during the early post-transplant period when baseline renal allograft function is not well-established. They can, however, be considered when there is a specific indication for their use, such as proteinuria and posttransplant erythrocytosis. Rising serum creatinine from ACEI or ARB therapy, although reversible, is the main reason they are avoid. This change is serum creatinine can be difficult to differentiate from other causes of worsening renal allograft function, particularly acute renal allograft rejection, leading to unnecessary workups and possibly unnecessary renal allograft biopsies. Similarly to ACEIs and ARBs, diuretics are not generally used as the first line for BP control in kidney transplant recipients. It may be used for volume control at immediate or early post-transplant.

From the characteristics, clinical outcomes, and side effect profiles of antihypertensive medications, we routinely use dihydropyridine calcium channel blockers and/or beta-blockers as first-line antihypertensive medications. Since the majority of patients do not achieve BP control during the immediate or early post-transplant period, the combination of dihydropyridine channel blockers with beta-blockers is commonly used in our transplant center. Once renal allograft function is established and stable, we consider adding or replacing ACEI or ARB for dihydropyridine channel blockers and beta-blockers if there are appropriate indications, such as proteinuria or post-transplant polycythemia.

Table 3 summarizes characteristics of the most commonly used antihypertensive medications and rationales for selecting each BP medication in kidney transplant recipients (154, 175, 176, 198–204).

**Table 3:** Summarized common antihypertensive medications used in kidney transplant patients.

**Antihypertensive classes**	**Pros**	**Comments**	**Cons**	**Comments**
**Diuretics**				
Loop	- Generally, not the first line antihypertensive medication Used in CsA treated recipients - Used for volume control - May use with ACEI or ARB in TRAS	- Renal sodium excretion defect in CsA-induced HTN (198)	- Loop diuretic may worsen renal allograft function from redistribution of decreased renal blood flow at juxtamedullary cortex and outer medulla (199) and ↓ oxygenation in medulla due to decreased cortical vascular resistance diverting medullary perfusion (200).	
			- Electrolyte disturbance	- Hyponatremia- Hypomagnesemia- Hyperuricemia
Thiazide	- May consider in hypomagnesemic patients who needs volume control from diuresis (154).	- Not the first line antihypertensive medication	- Potential volume depletion - Hyperlipidemia	
	- May consider in salt-sensitive HTN from CNIs	- WNK-SPAK-NCC pathway	- Electrolyte disturbance	- Hyponatremia - Hypomagnesemia
	- May use with ACEI or ARB in TRAS			- Hyperuricemia
CCB	-May improve renal allograft function (201) and lower DGF but inconclusive	-Afferent arteriolar vasodilatation (202)	-Non-dipyridamole CCB is CYP450 inhibitor and increases CNI level	-CYP450 3A4 enzyme inhibitor → ↑CNI and mTOR inhibitors level
ACEI/ARB	- Anti-proteinuric		- Hyperkalemia	
	- Cardioprotection (203, 204)		- Anemia	
	- May use with diuretic in TRAS		- Elevated creatinine	- In the setting of volume depletion, TRAS
Beta-blockers	-For cardioprotection		- Mask symptoms of hypoglycemia and thyrotoxicosis - Worsening lipid profiles	
			- Hyperkalemia	-Especially with mTOR inhibitors
			- Potential rebound HTN	
Mineralocorticoid receptor antagonists	-Systolic dysfunction	-Safe with using ACEI and ARB	-Hyperkalemia	
Alpha_1_ antagonist	- Comparable to ACEI for BP control - Generally, not the first line antihypertensive agents	- May need to add other antihypertensive agents		
Alpha_2_ agonist	- Lower plasma renin activity that modulated renal vascular resistance and subsequently lower MAP (140). - No change in GFR and effective renal plasma flow (141)		-Potential rebound HTN	-Need to be slowly tapered off if medication discontinuation is needed.

*ACEI, angiotensin-converting enzyme inhibitors; ARB, angiotensin receptor blockers; CCB, calcium channel blockers; CNI, calcineurin inhibitor; CYP, cytochrome; DGF, delayed graft function; GFR, glomerular filtration rate; mTOR, mammalian target of rapamycin; TRAS, transplant renal artery stenosis; WNK-SPAK-NCC, WNK, With-No-K(Lys)—STE20/SPS1-related proline/alanine-rich kinase—Sodium Chloride Cotransporter*.

## Blood Pressure Guideline for Kidney Transplant Recipients

BP targets have been a controversial topic not only in the non-transplant population but also in kidney transplant recipients. Several professional societies from different countries have created clinical practice guidelines with some similarities and differences (Table 4) (26, 27, 205–212).

**Table 4:** Summarized blood pressure guideline for kidney transplant recipients from different scientific medical societies.

	**Recommended BP target (mmHg)**	**Recommended BP target in special circumstances**	**Recommended first line antihypertensive medications**	**Comments**
KDIGO 2012 (205)	≤ 130/80 (2D)		- Time after transplantation - Use of calcineurin inhibitors - Albuminuria - Comorbidity (not graded)	
K/DOQI 2004 (206)	≤ 130/80		- Insufficient data to recommend any class of antihypertensive medications	-Integrate non-pharmacological managements including weight loss, dietary sodium restriction, smoking cessation
K/DOQI 2012 (commentary on KDIGO 2012) (207)	≤ 140/90		-Individualized choice of antihypertensive agents	
AST 2009 (208)	≤ 130/80 in adult ≥18 years old (2C)		- No preferred choice of antihypertensive medication - ACEIor ARB if urine protein ≥1 g/day for adult ≥18 years old *(Not graded)*	
BRA 2011 (209)	Clinic blood pressure ≤ 130/80	125/75 mmHg if urine protein/creatinine ratio (PCR) >50 or ACR>35) (2C)	- RAS may be more effective in the minimization of proteinuria but	- Used with caution in the first 3 months post-transplant (2C)
CSN Work Group 2014 (comment on KDIGO 2012) (210)	≤ 140/90 regardless of the level of albuminuria	≤ 130/80 in kidney transplant recipients with diabetes	- Based on comorbidities including DM, stroke, CAD, CCB, recent MI, and CHF - RAS blockers should be avoided in the immediate post-transplantation	
ERBP Work Group 2013 (211)	≤ 130/80		- ACEI and ARB should be avoided in the first month post-transplant - CCB especially non-hydropyridine CCB may interact with CNI and m-TOR inhibitors.	- Potential confounding of rising serum creatinine on acute rejection
KHA-CARI 2012 guideline (adaptation of the 2009 KDIGO) (212)	≤ 130/80 in adult	-Tighter BP control with BP <125/75 in the patient with proteinuria >1 g/day (2C)	-Suggests using CCB as the first line antihypertensive agent; however, this should be balance with the patients' comorbidity and proteinuria. Closely monitor CNI level.	
2017 ACC/AHA (26, 27)	<130/80 mmHg	Reasonable BP target	- CCB is reasonable agent due to improved GFR and kidney survival	

*() denotes the strength of recommendation ACC/AHA, American College of Cardiology/American Heart Association; AST, American Society of Transplantation; BP, blood pressure; BRA, British Renal Association; CAD, coronary artery disease; CCB, calcium channel blockers; CHEP, Canadian Hypertension Education Program; CHD, congestive heart failure; CSN, Canadian Society of Nephrology; DM, diabetes mellitus; ERBP, European Renal Best Practice; KDIGO, Kidney Disease: Improving Global Outcomes; K/DOQI, Kidney Disease Outcomes Quality Initiative; KHA-CARI, Kidney Health Australia-Caring of Australians with Renal Impairment; RAS, renin-angiotensin system*.

Until there is strong clinical outcome evidence in terms of CV, patients, or renal allograft survival, BP targets should be individualized, taking into account immunologic and non-immunologic factors that are contributing to HTN in each kidney transplant recipient.

## Other Management for Blood Pressure

### Native Nephrectomy

In ESRD patients with resistant HTN, defined as uncontrolled BP with at least three antihypertensive medications of which one is a diuretic, secondary HTN should be considered. Apart from renovascular and hormonal causes of secondary HTN, failed kidneys can contribute to HTN or be the cause of uncontrolled HTN in ESRD patients. In ESRD secondary to ADPKD, the mechanism of HTN from failed kidneys is related to intra-renal renin instead of systemic renin (213, 214). In kidney transplantation, the presence of failed native kidneys is associated with post-transplant resistant HTN. This is presumed in part related to the effect of angiotensin II (215). In these kidney transplant recipients with resistant HTN, ACEIs and native nephrectomy may need to be considered.

There is evidence that kidney transplant recipients with pre- or post-transplant native nephrectomy have decreased BP when compared to those without native nephrectomy (Table 5) (216–221). The majority of these studies were notably conducted in patients with ADPKD as the cause of ESRD.

**Table 5:** Studies examining effectiveness of native nephrectomy and blood pressure control in kidney transplant recipients.

**References**	***n*/age**	**Exposure**	**Outcomes**	**Other outcomes**	**Conclusions**
Vanrenterghem et al. (216)	707 first DDRT on CsA	Group I: 264 patients with post-transplant bilateral native nephrectomy Group II: 443 patients with non-nephrectomy	Proportion of patients requiring antihypertensive medication was lesser in group I compared to group II at 1-year post-KTx [45.3 vs. 65.8% (*P* < 0.0001)]. Group I had lower DBP than group II (83 ± 10 vs. 87 ± 25; *P* < 0.02). Lower proportion of patients with Hb >17 g/dl in group I compared to group II [6 (2.3%) vs. 44 (9.9%); *P* < 0.0001] during the first year post-KTx. The directions of these associations were not different 5 years after transplantation.	Patient and graft survivals and renal allograft function were not different between two groups. Proportion of acute rejection during the first year post-KTx was lower in group I than that in group II [0.62 ± 0.88 vs. 0.78 ± 1.02 (*P* = 0.0285)]	Posit-transplant arterial HTN and erythrocytosis could be controlled by post-transplant bilateral native nephrectomy.
Lerman et al. (217)	5 patients 2 with HTN or FSGS had KTx-−3 with DM had KPx/age range 49–69 years old	Post-transplant laparoscopic bilateral native nephrectomy	↓MAP in all 5 patients at 3–6 months post nephrectomy. ↓The number of antihypertensive medications in 4 patients.	Renal allograft functions - Stable in 3 patients- ↓ in 2 patients.	Benefit of nephrectomy (e.g., improved BP, decreased or no increased number antihypertensive medications, and stable renal allograft function) in 3 patients with a lower baseline creatinine at the time of bilateral native nephrectomy.
Iino et al. (218)	50 ADPKD patients with KTx	Group I: 24 patients with no native nephrectomy, Group II: 7 patients with pre-transplant unilateral native nephrectomy Group III: 19 patients with pre-transplant bilateral native nephrectomy	Proportions of HTN 6 months post-KTx compared to those pre-KTx were lower in group III, but higher in group I and II (the hypertension rate before and within 6 months after transplantation was I-70.8 vs. 82.6%, II-71.4 vs. 80%, and III-78.9 vs. 61.1%, respectively). Prevalence of HTN after 6 months post-KTx was highest in group I followed by group III and then group II [HTN after 6 months was more prevalent in I group (95.5%) than in II (40%)], III (64.7%) groups (*p* = 0.008)	Graft failure and proteinuria were not significant differences among three groups.	ADPKD patients post-KTx without native nephrectomy had higher prevalence of post-Tx HTN than those with pre-KTx unilateral or bilateral native nephrectomy.
Shumate et al. (219)	118 ADPKD patients undergoing KTx from 2003 to 2013/mean age 53.6 ± 10.3 years	Group I: 54 patients with KTx alone Group II: 32 patients with simultaneous ipsilateral native nephrectomy-KTx Group III: 32 patients with simultaneous ipsilateral native nephrectomy-KTx and delayed contralateral native nephrectomy	The quantity and daily dose of antihypertensive medications were lower in group II compared to group I at 12, 24, and 36 months post-KTx. Mean number of antihypertensive medications from post-ipsilateral nephrectomy to 12 months post-contralateral nephrectomy was lower in group III compared to non-nephrectomy group.		Quantity and defined daily dose of antihypertensive medications were significant lower in simultaneous ipsilateral native nephrectomy-KTx and even lower in delayed contralateral native nephrectomy.
Jo et al. (220)	42 ADPKD patients with KTx/mean age: Group I 50.2 ± 10.4 years vs. Group II 52.4 ± 8.6 years	Group I: 26 ADPKD with simultaneous native nephrectomy-KTx Group II: 16 ADPKD patients with KTx-alone	Group I required less antihypertensive medications compared to group II at 1, 3, 6, and 12 months post-KTx.	Group I had significant higher proportion of peri-operative hypotension compared to group II (69.2 vs. 37.5%, *p*-value 0.045).	Patient and renal allograft survivals and renal allograft function were not different between two groups.
Obremska et al. (221)	32 patients with pretransplant bilateral native nephrectomy/mean age 51.72 ± 14.46 vs. 51.94 ± 12.97 (control)	Group I (study group): 32 patients with pretransplant bilateral native nephrectomy Group II (control group): healthy patients Median time to follow-up: - Group I 95 months (54.5, 115.5) -Group II 91 months (57.5–132.4).	Group I had lower SBP and the number of antihypertensive medications compared to group II.	Group I had lower LVMI, LAVI, and left ventricular mass evaluated by CMR. Group I had milder LVDD than group II.	

*ADPKD, autosomal dominant polycystic kidney disease; CMR, cardiac magnetic resonance; CsA, cyclosporine A; DBP, diastolic blood pressure; Hb, hemoglobin; KPx, kidney-pancreas transplantation; KTx, kidney transplantation; LAVI, left atrial volume index; LVDD, left ventricular diastolic dysfunction; LVMI, left ventricular mass index; MAP, mean arterial pressure*.

### Native Renal Sympathetic Denervation

Apart from the angiotensin II pathway, sympathetic overactivity from failed native kidneys is another mechanism that leads to resistant HTN. Renal and systemic sympathetic hyperactivity contributes to the pathophysiology of resistant HTN. The effect of RDN on BP control was established (222). In kidney transplant recipients with native non-functioning kidneys, sympathetic nervous system activation from afferent signal of native kidneys was preserved (223).

### Surgical Renal Denervation by Bilateral Native Nephrectomy

Complete RDN can be performed by bilateral native nephrectomy. A retrospective study in 32 kidney transplant recipients undergoing pre-transplant bilateral native nephrectomy revealed lower SBP, a number of antihypertensive medications, left ventricular mass index, and left atrial volume index but higher left ventricular diastolic dysfunction compared to a matched (age, gender, creatinine levels, eGFR, immunosuppressive treatment, and the time of renal replacement therapy) control group (221). This data supports the effect of RDN as treatment options for HTN in this population.

Although native nephrectomy can improve post-transplant HTN, both pre- and post-transplant native nephrectomy can lead to surgical complications, which can cause impaired renal allograft function. Therefore, native nephrectomy for the purpose of BP control in kidney transplant recipients should be performed in select patients with severely uncontrolled post-transplant HTN whom, without treatment, would have deterioration of renal allograft function or be at increased risk of CV complications. Patients who undergo bilateral native nephrectomy for alternative indications such as recurrent infection, discomfort from large polycystic kidneys, or suspicious native renal tumors may have the added benefit of improved BP control.

### Catheter Ablative Renal Denervation

Partial reinnervation of the human transplanted kidney has been shown histologically. Axonal regeneration in a human transplant kidney started at 28 days to 5 months post-kidney transplantation and was complete within 8–12 months to 2 years (224, 225). The function of efferent renal nerves that reinnervate transplanted renal arteries, however, may not be the same as what occurs in native renal nerves (226, 227). One study of renal hemodynamics, sodium excretion, and tubular function after noradrenaline infusion (2 μg h^−1^ kg^−1^) and lower body negative pressure (−27 mmHg) in 25 kidney transplant recipients and 10 normal subjects concluded that the reinnervated efferent renal nerves in the human transplanted kidney are still essentially functionally denervated (228). Since complete RDN by bilateral native nephrectomy in kidney transplant recipients is invasive and carries a risk of additional operation, native RDN is another option, and it should be reserved for selected patients.

Two reported kidney transplant recipients with resistant HTN had improved BP control after RDN of native kidneys (229, 230). An investigator-initiated, prospective, single-center RCT with 6-month follow-up of 18 kidney transplant recipients with resistant HTN compared the feasibility and efficacy of renal sympathetic denervation as compared to medical therapy alone. The former group had a significantly decreased office SBP of 23.3 ± 14.5 mmHg and a higher proportion of patients who converted from non-dippers to dippers. Nocturnal BP recorded with 24-h ABPM, but not daytime BP, was also lower by 10.38 ± 12.8 mmHg in the RDN group, though this was not statistically significant. Safety endpoints including changes in renal allograft function and renovascular complications were not different between the 2 groups (231). Although native RDN is effective in controlling BP, larger RCTs with a sham-control are required.

## Conclusions

HTN is a very common disease in CKD and ESRD and remains so after kidney transplantation. The pathogenesis of post-transplant HTN is complex. BP measurement is still the main barrier to accurately diagnose and follow-up in HTN management. A 24-h ABPM, though the gold standard, is inconvenient and not wildly utilized. The choice of antihypertensive medication requires the clinician to take transplant and immunological factors into the consideration. Management for OSA and interventions for resistant HTN, such as transplant renal artery angioplasty ± stenting, bilateral native nephrectomy, and native RDN, remain options for resistant HTN in selected kidney transplant recipients. There is no conclusive BP target for this population and therapy targets need to be individualized. Further research to develop a stronger body of evidence for the pathogenesis of post-transplant HTN and guide clinicians on the appropriate BP measurement method, use of antihypertensive medications, surgical or procedural interventions, and establish BP targets for kidney transplant recipients.

## Author Contributions

ET participated in designing the topics and detail of the manuscript, writing the manuscript, and preparing figures and tables. MM participated in the design of the topics, detail of the manuscript, and the editing of the manuscript. BH, UR, DD, HI, and AA participated in preparing and reviewing the manuscript. AF and RH participated in preparing, editing, and reviewing the manuscript. KK-Z participated in designing the topics and detail of the manuscript as well as preparing and reviewing the manuscript. All authors contributed to the article and approved the submitted version.

## Conflict of Interest

The authors declare that the research was conducted in the absence of any commercial or financial relationships that could be construed as a potential conflict of interest.

## Abbreviations

24-h ABPM: 24-hour ambulatory blood pressure monitoring
AVF: arteriovenous
ACC/AHA: American College of Cardiology/American Heart Association
ACEI: angiotensin-converting enzyme inhibitors
ADPKD: autosomal dominant polycystic kidney disease
AHI: apnea-hypopnia index
ARB: angiotensin II receptor blockers
ATI: acute tubular injury
AVF: arteriovenous fistula
BMI: body mass index
BP: blood pressure
CAD: coronary artery disease
CAN: chronic allograft nephropathy
CCB: calcium channel blockers
CDU: color Doppler ultrasonography
CI: confidence interval
CIN: contrast-induced nephropathy
CKD: chronic kidney disease
CMR: cardiac magnetic resonance
CMV: cytomegalovirus
CNI: calcineurin inhibitor
CNS: central nervous system
CO_2_: carbon dioxide
Cr: creatinine
CrCl: creatinine clearance
CsA: cyclosporine A
CT: computed tomography
CV: cardiovascular
CVD: cardiovascular diseases
CYP: cytochrome P (CYP)
DBP: diastolic blood pressure
DCT: distal convoluted tubule
DDRT: deceased donor renal transplantation
ESRD: end-stage renal disease
FGF23: fibroblast growth factor 23
FHHt: familial hyperkalemic hypertension
FST: furosemide stress test
HBPM: home blood pressure monitoring
Hb: hemoglobin
HFpEF: heart failure with preserved ejection fraction
HTN: hypertension
IV: intravenous
KPx: kidney-pancreas transplantation
KTx: kidney transplantation
LAVI: left atrial volume index
LVDD: left ventricular diastolic dysfunction
LVH: left ventricular hypertrophy
LVMI: left ventricular mass index
MAP: mean arterial pressure
MCRA: mineralocorticoid receptor antagonist
MD: mean difference
MHC: major histocompatibility complex
MI: myocardial infarction
MRA: magnetic resonance angiography
mTOR: mammalian target of rapamycin
NaCl: sodium chloride
Na-K-ATPase: sodium-potassium adenosine triphosphase
Na-K-2Cl: sodium-potassium-2 chloride
NCC: sodium chloride cotransporter
OBP: office blood pressure
OSA: obstructive sleep apnea
PAMORA: Peripherally acting mu opioid receptor antagonists
PDGF: platelet-derived growth factor
PSV: peak systolic velocity
RAAS: Renin-Angiotensin-Aldosterone System
RAS: renal artery stenosis
RCT: randomized controlled clinical trial
RDN: renal denervation
RI: resistive index
ROMK: renal outer medullary potassium
RRT: renal replacement therapy
SAW: steroid avoidance or withdrawal
SBP: systolic blood pressure
SLEPT: SLeep disorders Evaluation in Patients after kidney Transplantation
SPAK: STE20/SPS1-related proline/alanine-rich kinase
TRAS: transplant renal artery stenosis
WNK: With-No-K(Lys).
